# Interaction between the C-terminal region of human myelin basic protein and calmodulin: analysis of complex formation and solution structure

**DOI:** 10.1186/1472-6807-8-10

**Published:** 2008-02-19

**Authors:** Viivi Majava, Maxim V Petoukhov, Nobuhiro Hayashi, Päivi Pirilä, Dmitri I Svergun, Petri Kursula

**Affiliations:** 1 Department of Biochemistry, University of Oulu, Oulu, Finland; 2 EMBL Hamburg Outstation, Hamburg, Germany; 3 Institute of Crystallography, Russian Academy of Sciences, Moscow, Russia; 4 Institute for Comprehensive Medical Science, Fujita Health University, Japan

## Abstract

**Background:**

The myelin sheath is a multilamellar membrane structure wrapped around the axon, enabling the saltatory conduction of nerve impulses in vertebrates. Myelin basic protein, one of the most abundant myelin-specific proteins, is an intrinsically disordered protein that has been shown to bind calmodulin. In this study, we focus on a 19-mer synthetic peptide from the predicted calmodulin-binding segment near the C-terminus of human myelin basic protein.

**Results:**

The interaction of native human myelin basic protein with calmodulin was confirmed by affinity chromatography. The binding of the myelin basic protein peptide to calmodulin was tested with isothermal titration calorimetry (ITC) in different temperatures, and K_d _was observed to be in the low μM range, as previously observed for full-length myelin basic protein. Surface plasmon resonance showed that the peptide bound to calmodulin, and binding was accompanied by a conformational change; furthermore, gel filtration chromatography indicated a decrease in the hydrodynamic radius of calmodulin in the presence of the peptide. NMR spectroscopy was used to map the binding area to reside mainly within the hydrophobic pocket of the C-terminal lobe of calmodulin. The solution structure obtained by small-angle X-ray scattering indicates binding of the myelin basic protein peptide into the interlobal groove of calmodulin, while calmodulin remains in an extended conformation.

**Conclusion:**

Taken together, our results give a detailed structural insight into the interaction of calmodulin with a C-terminal segment of a major myelin protein, the myelin basic protein. The used 19-mer peptide interacts mainly with the C-terminal lobe of calmodulin, and a conformational change accompanies binding, suggesting a novel mode of calmodulin-target protein interaction. Calmodulin does not collapse and wrap around the peptide tightly; instead, it remains in an extended conformation in the solution structure. The observed affinity can be physiologically relevant, given the high abundance of both binding partners in the nervous system.

## Background

The myelin sheath is a tightly packed multilamellar membrane structure crucial for the correct functioning of the vertebrate nervous system. Myelin carries a specific set of proteins, whose expression is tightly regulated during development. Biochemically, the composition of myelin in the central and peripheral nervous system (CNS and PNS, respectively) is different from each other [1]. Mutations in myelin proteins or an autoimmune attack towards them can lead to devastating neurological diseases.

One of the most abundant proteins of myelin is the myelin basic protein (MBP) [2,3]. MBP is a protein family, of which the 18.5-kDa isoform predominates in adult myelin [2,4]. In CNS myelin, it comprises 30% of the total protein; it is also present in PNS myelin [5]. MBP is thought to be involved in the tight association of the cytoplasmic leaflets of apposing myelin membranes within compact myelin, where there is little, if any, cytoplasm present [6]. Several segments of MBP are target autoantigens that have been characterised in multiple sclerosis [7].

A bewildering amount of post-translational modifications, in addition to extensive alternative splicing, have been observed for MBP, leading to a number of size and charge isoforms [2]. MBP has also been characterised as being intrinsically unstructured, with a possibility of local folding, especially upon binding to ligands [3]. A low-resolution 3-dimensional model for MBP adsorbed to a lipid monolayer has been built based on electron microscopy [8,9]. Solution scattering experiments have also indicated an unfolded structure for lipid-free MBP; in the lipid-bound state, however, the protein seems compact but not globular [10].

Several interaction partners for MBP have been characterised, including actin [11-13], tubulin [14,15], and calmodulin (CaM) [11,16-23]. Although the interaction between MBP and CaM was initially reported already in 1980 [17], relatively little structural information is available about the interaction [22]. In addition, MBP seems to have multiple regions capable of binding CaM [21,22], and it is not fully clear which of the CaM-binding sites are of physiological relevance. Some assays have also indicated a heterogeneous mode for the interaction [20,21], and the interaction is affected by MBP post-translational modifications, such as citrullination [20-22,24]. The main CaM-binding site has been suggested to reside in the C-terminal region of CaM, between residues 132–167 [20,21]. At least one more site lies in the central/N-terminal region of MBP [20-22].

In the present study, we have used a synthetic peptide from the predicted CaM-binding segment near the C-terminus of human MBP to study the MBP-CaM interaction. We have used a number of biochemical and biophysical methods to confirm the interaction, thus accurately mapping the binding site of the peptide in CaM, and to obtain thermodynamic and structural data regarding complex formation in solution.

## Results

### Native human brain MBP binds to CaM

**Figure 1:**
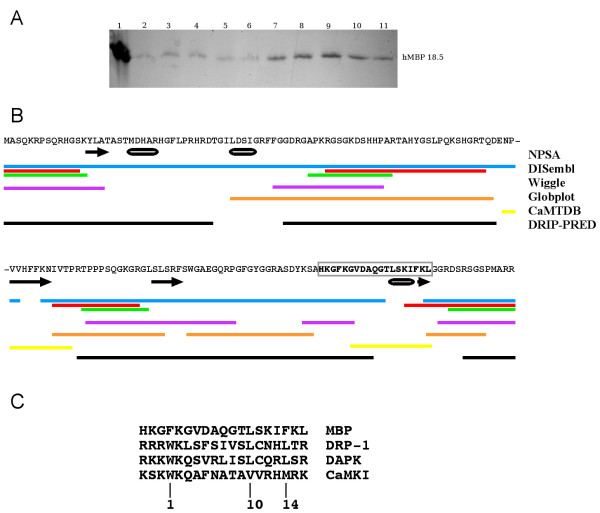
**Interaction between human brain MBP and CaM and selection of the peptide for the CaM interaction study**. A. Electrophoretic analysis by SDS-PAGE of the fractions from affinity chromatography of human brain MBP on CaM-sepharose. The samples are as follows: 1, adult human brain MBP isoforms loaded into the column; 2–6, washes with a buffer containing 50 mM HEPES pH 7.5, 150 mM NaCl, and 2 mM CaCl_2_; 7–11, elution with a buffer containing 50 mM HEPES pH 7.5, 150 mM NaCl, and 2 mM EGTA. The observed strong protein band corresponds to the 18.5-kDa main human MBP isoform. The presence of also the 17.2-kDa isoform in both the second wash (lane 3) and the second EGTA eluate (lane 8) was confirmed by mass spectrometry (see Additional file 1). B. The sequence of human 18.5-kDa MBP, in which the bold, boxed region is the peptide eventually selected. Below the sequence, various results from sequence analysis are shown: NPSA secondary structure prediction, black arrows (helix) and cylinders (strand); DISembl disorder prediction using the 3 different learning sets in the program, blue-red-green; Wiggle prediction for functionally flexible regions, magenta; GlobPlot prediction of disorder, orange; suggested CaM-binding sites according to the CaM target database, yellow; DRIP-PRED disorder prediction, black. C. Manual alignment of the selected peptide with CaM-binding segments from three human CaM-dependent protein kinases. The positions of the conserved hydrophobic anchor residues in CaM-dependent kinases are indicated below the alignment.

Affinity chromatography on CaM-sepharose was used to investigate the binding of human brain native MBP by CaM. The results clearly indicated that in the presence of calcium ions, MBP is retained in the affinity matrix, and that upon complexation of calcium by using EGTA, MBP is released (Figure 1A). Thus, the interaction is calcium-dependent, as earlier shown for bovine, murine, and human brain MBP [17-20]. Mass spectroscopic analysis indicated that the main human adult MBP isoforms, 17.2 and 18.5 kDa, were both present in the EGTA eluate, indicating that neither one of them is deficient in binding CaM (Additional file 1). Both in gel electrophoresis and mass spectroscopy, the 18.5-kDa isoform was more abundant.

### Selection of a putative CaM-binding peptide

Previously, a region near the C-terminus of MBP has been suggested to be its major CaM-binding site [20,21]. We used a number of sequence analysis methods to select a peptide from this region that would be expected to bind CaM with high affinity. A combination of the following parameters was used: predicted propensity to fold into a helix, a low score in disorder predictions, the presence of a putative CaM-binding site according to the CaM target database [25], and detectable sequence homology to other well-characterised CaM targets. In addition, a search for putative functionally flexible regions was carried out using Wiggle. The results of these analyses are shown in Figure 1.

The peptide HKGFKGVDAQGTLSKIFKL, corresponding to residues 138–156 of human 18.5-kDa MBP, was selected for the experiments. In brief, this is one of the two regions recognised as putative CaM targets by the CaM target database, and clearly one of the few regions in MBP that are consistently not predicted to be disordered by various disorder prediction algorithms. It is also predicted to be able to fold into an α-helix.

### Conformational analysis by circular dichroism spectroscopy

**Figure 2:**
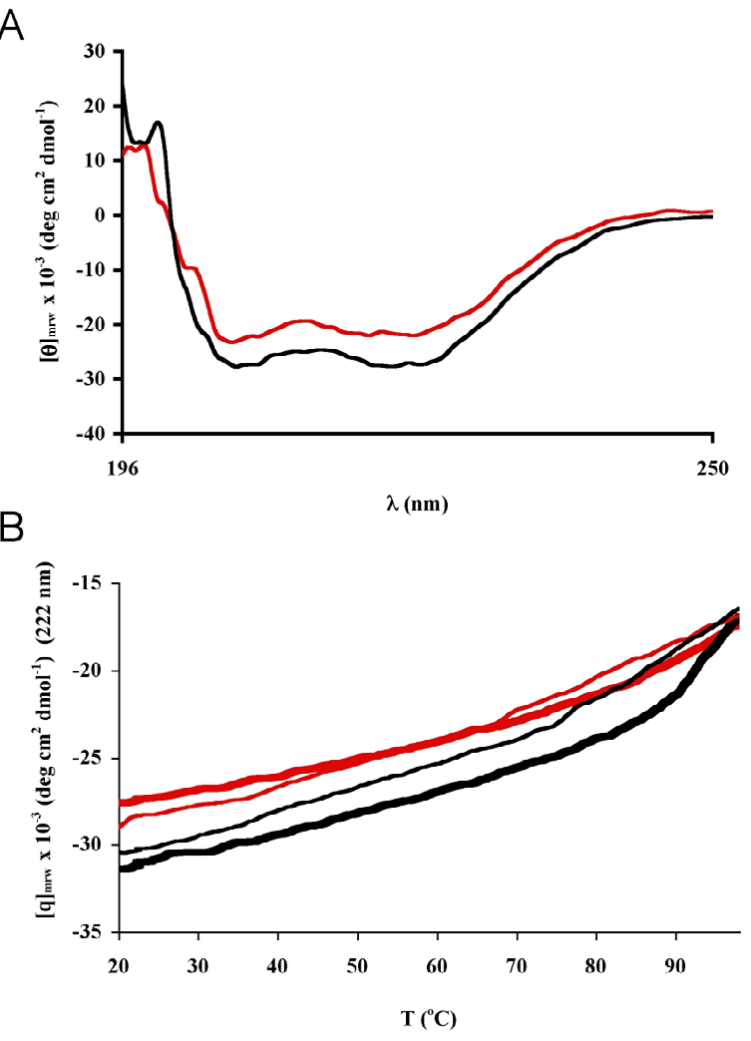
**Circular dichroism spectroscopy**. A. CD spectra of the CaM-peptide complex (red) and CaM (black). CaM was used at 0.050 mg/ml (in both samples) and the peptide at 0.030 mg/ml. B. Thermal stability curves of CaM (black) and the complex (red), monitored at 222 nm. Mean residue ellipticity as a function of temperature was measured during heating from +20 to +98°C (thick lines). Refolding of the samples during cooling from +98 to +20°C is indicated by the thin lines.

Circular dichroism (CD) experiments were performed to compare the secondary structure, folding, and thermal stability of CaM in the presence and absence of the MBP peptide. The CD spectrum of CaM alone showed a shape typical of α-helical proteins, with negative minima at 206 and 221 nm and a positive maximum at 196 nm (Figure 2A). The CD spectrum of the complex was only slightly different from the spectrum of CaM, indicating that no major conformational changes take place upon complex formation. The k2d program [26] was used to predict the secondary structure contents (Table 1). The proportional content of α-helix was somewhat smaller in the complex than in CaM, reflecting the observation that the peptide in the absence of CaM contained no α-helix under the employed conditions (Table 1). While the fitting method suggests a large fraction of β-strand in the peptide in the absence of CaM, this reflects, rather, the inadequacy of the method for calculating the structure of a short peptide (or an unfolded protein), which is mostly unfolded, and probably contains a mixture of transient structures. For the structure modeling (see below), the peptide was modeled as a helix due to its homology to other (kinase) peptides that always bind CaM in a helical conformation (but are indeed disordered when not bound).

**Table 1:** Deconvolution of the CD spectra into secondary structure content, using k2d. The results obtained by subtracting the CaM and peptide spectra from the complex spectrum are shown on the last two rows.

Sample	α-Helix	β-Sheet	Random coil
CaM	0.88	0	0.12
peptide	0.05	0.47	0.48
Complex	0.72	0.03	0.25
Complex-CaM	0.03	0.50	0.47
Complex-peptide	0.72	0.03	0.26

A thermal scan from +20 to +98°C demonstrated that the complex as well as CaM is thermally very stable, showing the presence of folded structure even at temperatures exceeding +95°C (Figure 2B). A major transition, increasing the denaturation speed, occurs at a temperature of around +90°C for both samples. This transition is more pronounced in the absence of the peptide (CaM alone), which may indicate a stabilising effect by the peptide. Furthermore, cooling the samples back to +20°C in a similar fashion induced refolding of CaM (Figure 2C), and the presence of the peptide did not cause significant differences in the refolding.

### Calorimetric binding assay

**Figure 3:**
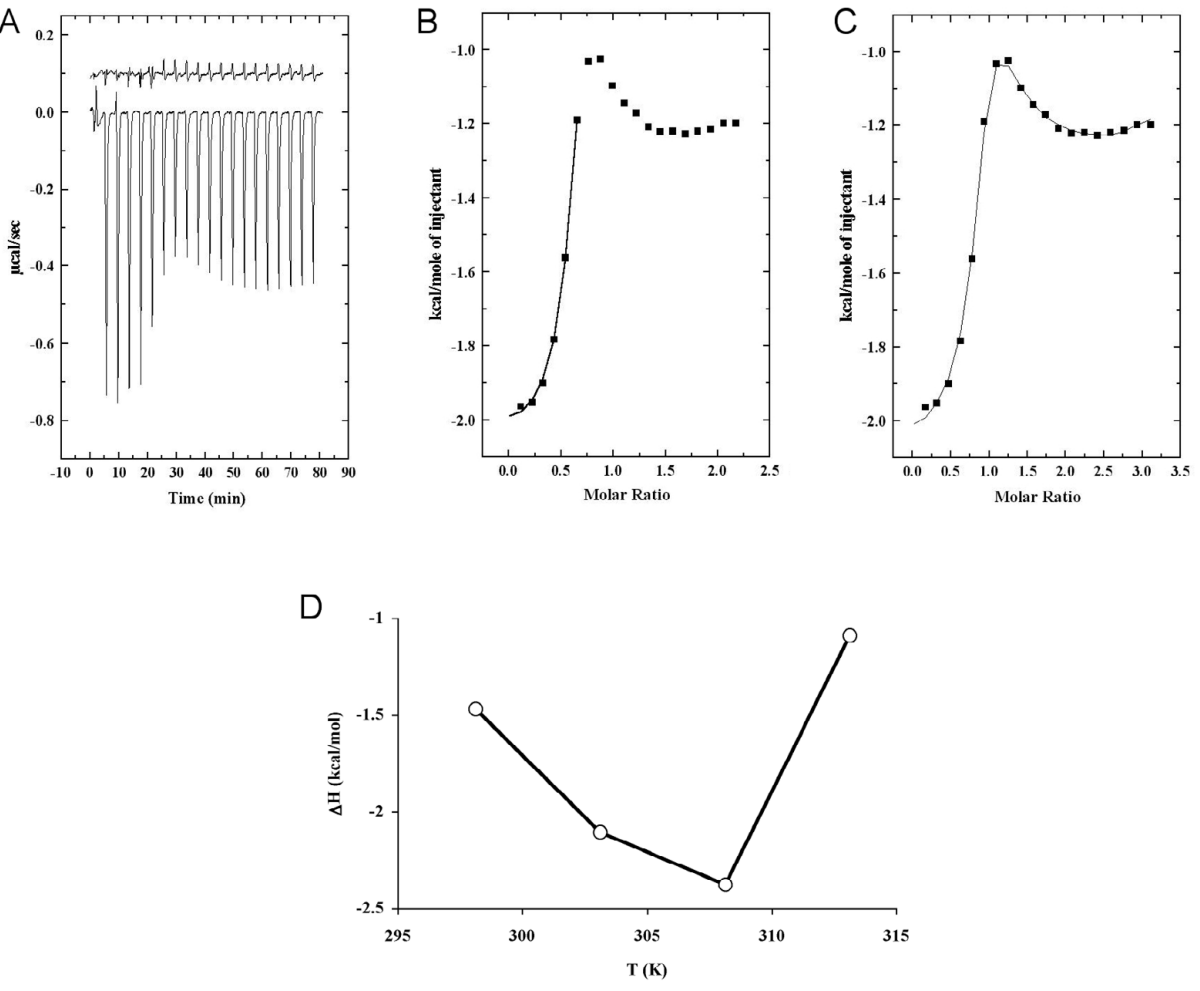
**Isothermal titration calorimetry**. A. Titration of the MBP peptide (1 mM) into a solution of CaM (0.1 mM) at +30°C. The results of a control reaction, where the MBP peptide was titrated into buffer at +30°C, are indicated above, moved up by 0.1 μcal/s for clarity. B. Fitting for the specific binding site only. The solid line indicates the non-linear least squares fit for the integrated area under peaks 2–7. The fitting model is one binding site. C. Fitting of all data to a multi-site model. The solid line indicates the non-linear least squares fit for the integrated area under peaks 2–20. The used model is for 4 sequential binding sites. Only the parameters for first binding event were used for analysis. D. The observed enthalpy as a function of temperature. The heat capacity (*ΔC*_*p *_= -0.13 kcal mol^-1 ^K^-1^) is the slope calculated by using *ΔH *values at temperatures +25 and +30°C.

Isothermal titration calorimetry was used to study the interaction between the MBP peptide and CaM (Figure 3). Experiments were done at four different temperatures (+25, +30, +35, and +40°C), and the calculated thermodynamic parameters are presented in Table 2. The K_d _values obtained were similar at temperatures below +35°C, and the heat-dependent conformational change of CaM – most likely slight unfolding of the central linker helix [27], starting around +35°C, can be seen in the measured ΔH values, that also become unreliable at +40°C while the affinity is decreased. The binding reaction has favourable enthalpy and entropy, being mainly entropically driven. The favourable entropy is, at least partially, a result of releasing ordered water into solution from the surfaces of the binding components.

**Table 2:** Thermodynamic parameters from ITC of the MBP peptide binding to CaM. The shown values are from fitting for the specific binding site only. In parentheses, the values for the first binding event are given when fitting is done to a 4-site model.

*T*	K_d _(μM)	*ΔH *(kcal/mol)	*-T ΔS *(kcal/mol)	*n*
298.15 K (25°C)	3.4 (1.0)	-1.47 ± 0.13 (-1.44 ± 0.04)	-6.00 (-6.73)	0.83 ± 0.23 (1)
303.15 K (30°C)	2.3 (1.1)	-2.11 ± 0.06 (-2.07 ± 0.02)	-5.71 (-6.18)	0.67 ± 0.02 (1)
308.15 K (35°C)	1.9 (1.0)	-2.38 ± 0.10 (-2.34 ± 0.03)	-5.67 (-6.13)	0.63 ± 0.02 (1)
313.15 K (40°C)	25.9 (1.7)	-1.09 ± 0.97 (-0.73 ± 0.05)	-5.48 (-7.56)	0.70 ± 0.17 (1)

The binding isotherms were characteristic of a biphasic binding event, in which first, a high-affinity specific binding occurs, and in the later stages of titration, a low-affinity nonspecific binding, with a large number of binding sites and favourable enthalpy, can be detected. The latter is probably due to electrostatic interactions between the peptide and CaM, after the single specific binding site is saturated. The measured dilution heat was basically zero (Figure 3A), and it was assumed that the titrations will each time approach zero.

To characterize the binding of the peptide to the specific binding site of CaM, only the peaks 2–7 were chosen for curve fitting to model 1:1 binding (Figure 3B). When using all data (data points 2–20), the best curve fitting could be obtained by using an artificial model implementing four sequential binding sites (Figure 3C). In both cases, the thermodynamic parameters for the first, high-affinity, binding event are highly similar (Table 2). This indicates that accurate values could be obtained for the high-affinity binding event even in the presence of non-specific background.

The effect of temperature on the enthalpy of binding, which can be reliably measured even in the presence of secondary low-affinity binding sites, is presented in Figure 3D. The enthalpies from the calorimetric studies were plotted against temperature and the formed slope (between 25–30°C) represents the heat capacity change *ΔC*_*p *_upon complex formation. The heat capacity, estimated from the two data points at +25 and +30°C, -0.13 kcal mol^-1 ^K^-1 ^(-0.54 kJ mol^-1 ^K^-1^), is negative for the complex. It is clear that more assays should be performed to obtain an accurate value for *ΔC*_*p *_of the complex.

### Surface plasmon resonance

**Figure 4:**
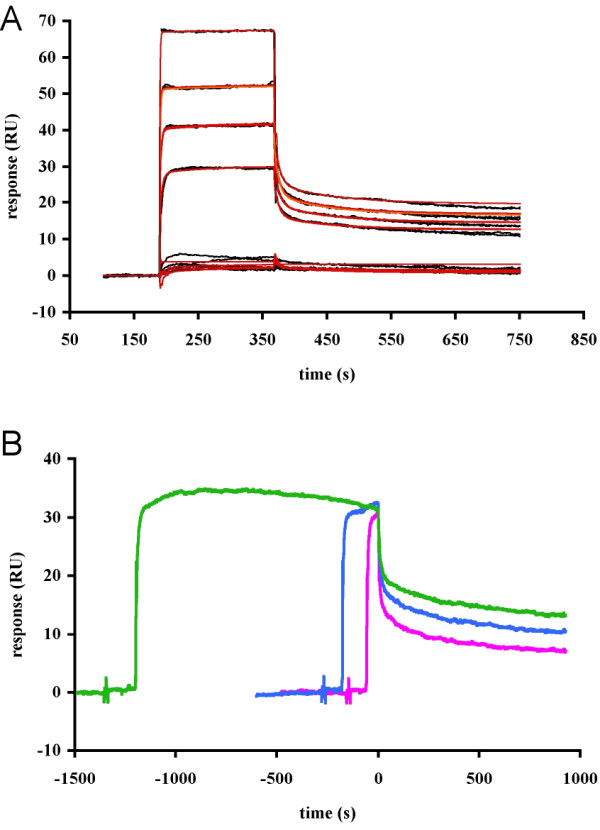
**Surface plasmon resonance**. A. SPR sensorgrams (black) of the binding of the MBP peptide (duplicate assays using 2.5, 5, 20, 50, 100, and 200 μM) onto immobilized CaM. Data were fit to the two-state conformational change model, and the resulting fit is shown in red. B. Control experiment for linked reactions (heterogeneity test) by SPR. The sensorgrams have been aligned to the end point of the injection. The injection times are as follows: 1 min (magenta), 3 min (blue), 20 min (green).

The interaction of the peptide and CaM was further studied by real-time biomolecular interaction analysis with surface plasmon resonance (SPR). The kinetics of the binding reaction were studied by injecting different concentrations of the MBP peptide over an immobilised CaM surface (Figure 4A). The data confirm the binding of the MBP peptide by immobilised CaM, and a K_d _value of 58 μM can be estimated from the data. The difference in the K_d _values obtained by SPR and ITC may be explained by the fact that CaM was immobilised on a solid support for the SPR analysis. The data could only be properly fit by a conformational change model (A+B <-> AB <-> AB*), in which, upon initial complex (AB) formation, the conformation of the complex alters (AB*). Even so, it became clear that the signal for the second conformation (AB*) is lower than for the first (AB), which significantly complicates the analysis and makes the calculated k_on _and k_off _values (k_a1 _= 6700 M^-1 ^s^-1^, k_d1 _= 0.14 s^-1^, k_a2 _= 0.004 s^-1^, k_d2 _= 0.011 s^-1^), especially for the second step of the reaction, unreliable. It is not possible to take such a situation into account in the BIAevaluation software, and in order to obtain a good fit, it was necessary to use additional parameters to describe the background level. A decrease in the SPR signal between two conformations has been observed previously for the binding of small molecules by a maltose-binding protein [28]. Ligand binding to a receptor that correspondingly decreases in hydrodynamic radius yielded a negative change in resonance units (RU). It was also proposed that the sign of the RU change is a function of the net change in hydrodynamic radius that occurs upon ligand binding [28]. Since by using the equilibrium RU values, the obtained Scatchard plot is curved (not shown), it is possible that similarly to the ITC experiment, the SPR signal is a combination of low- and high-affinity binding events. A further analysis of the data by non-linear regression by using a 2-site binding model (in GraphPad Prism) and the RU values at equilibrium gives evidence that a low-affinity binding event is seen at high concentrations of the peptide, and the corresponding K_d _values are approximately 10 and 1000 μM for the two binding events.

The conformational change model for the interaction was also suggested by the linked reaction control experiment, in which the dissociation rates of one analyte concentration (20 μM) after different injection times were compared (Figure 4B). An increased stability of the complex with longer injection times can be observed from the fact that the dissociation rates get slower with increased injection time. The experiment clearly showed that longer injections of the MBP peptide give a more stable CaM-peptide complex, which also suggests the occurrence of a conformational change. Furthermore, during longer injections, the signal decreases already during the injection, indicating that the signal for the AB* state is indeed lower than that for the AB state.

### Mapping the MBP-binding site of CaM by nuclear magnetic resonance (NMR) spectroscopy

**Figure 5:**
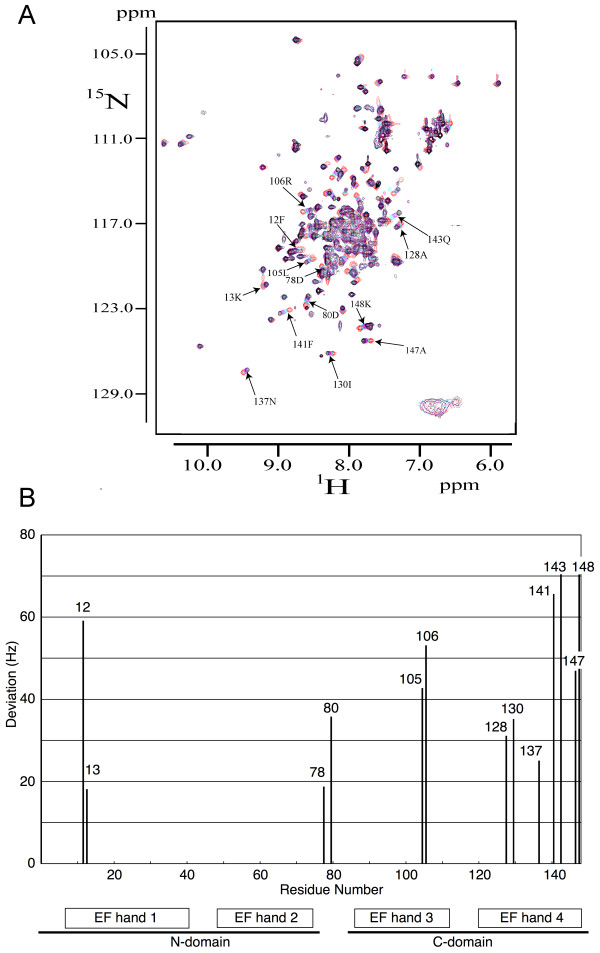
**^1^H-^15^N HSQC spectra for the titration of CaM with the MBP peptide**. A. Overlay of ^1^H-^15^N HSQC spectra of Ca^2+^/CaM alone (black) and the Ca^2+^/CaM-MBP peptide complex (pink; 1:0.333, blue; 1:0.666, red; 1:1). The samples contained 0.5 mM CaM, 120 mM NaCl, 2.5 mM CaCl_2_, and 50 mM deuterated Tris-HCl (pH 7.5) in 90% H_2_O and 10% D_2_O. Changes in cross-peak positions were quantified by [(Δ^15^N_Hz_)^2 ^+ (Δ^1^H_Hz_)^2^]^1/2^, and some cross-peaks showing larger shifts are indicated on the figure. B. Chemical shift perturbations quantified by [(Δ^15^N_Hz_)^2 ^+ (Δ^1^H_Hz_)^2^]^1/2^. Residues with large perturbations ([(Δ^15^N_Hz_)^2 ^+ (Δ^1^H_Hz_)^2^]^1/2 ^> 10.0) are shown. Numbers of each bar show their residual numbers. Positions of four EF hands are also indicated under the graph

NMR spectroscopy was used to identify CaM residues whose chemical shifts change upon forming the Ca^2+^/CaM-MBP peptide complex (Figure 5). To the most part, these residues are concentrated around the known peptide binding pocket of the CaM C-terminal lobe. Identified residues in this region include, for example, Leu105, Arg106, Ala128, Phe141, Gln143, Ala147, and Lys148.

The result that chemical shift perturbations were also observed for residues in the central helix indicates either bending of the helix upon peptide binding or a direct interaction between the peptide and the central helix. The chemical shift perturbations of some residues, mainly Phe12 and Lys13, of the N-terminal lobe suggest that the peptide is not only bound to the C-terminal lobe, but rather, interacts also weakly with the N-terminal lobe. Taken together, the NMR data show that the MBP peptide interacts most significantly with the hydrophobic pocket in the CaM C-terminal lobe, but also with parts of the central helix and the N-terminal lobe. These data were further used as restraints when building and refining a solution structure of the complex using small-angle X-ray scattering (SAXS) (see below).

### Solution structure from SAXS

#### Overall parameters

**Figure 6:**
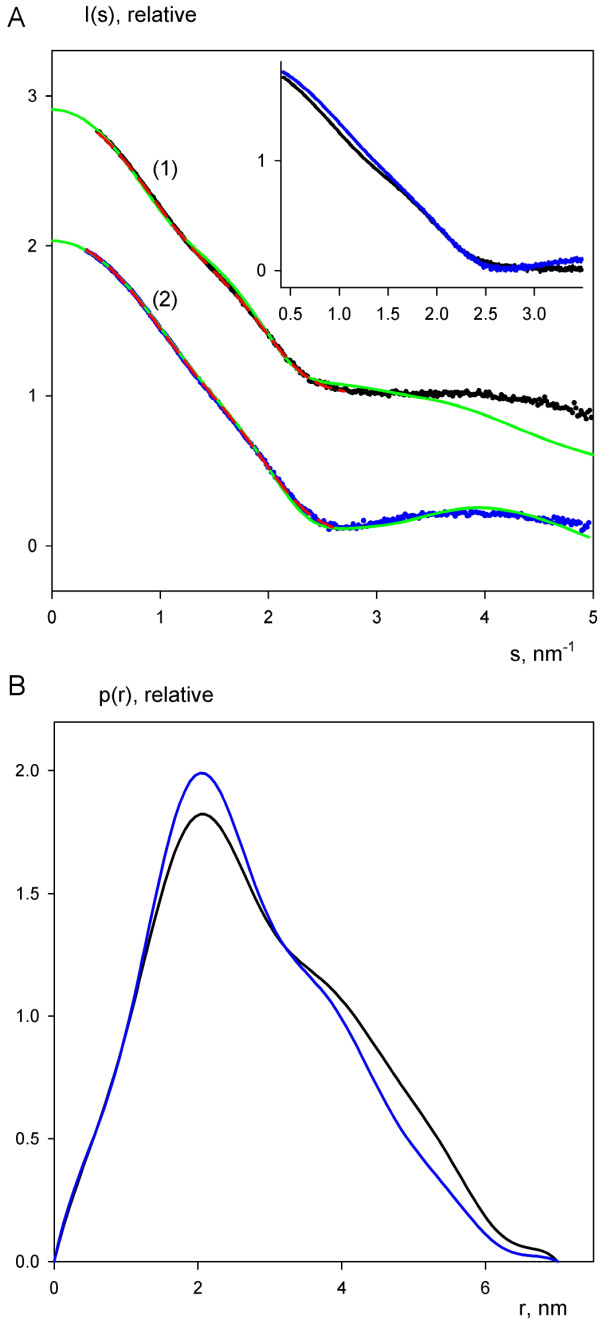
**Scattering profiles and distance distribution functions of free and loaded CaM**. A. Scattering data of CaM (1, black dots) and the complex (2, blue dots). Dots denote the experimental scattering data, the fits obtained by MONSA are displayed as red dashed lines, and the fits of the crystal structure of CaM and the rigid body model of the complex are shown as green solid lines. The plot displays the logarithm of the scattering intensity as a function of momentum transfer *s *= *4π sin(θ)/λ*, where *2θ *is the scattering angle and *λ *= 0.15 nm is the X-ray wavelength. The curves are arbitrarily displaced along the logarithmic axis for better visualization. The insert presents a direct comparison of the experimental curves without displacement. B. *p(r) *functions computed from the experimental X-ray scattering patterns using the program GNOM. The functions are presented in arbitrary units; the colouring scheme is the same that as used for the experimental data in (A).

The experimental scattering patterns from free CaM and the complex are presented in Figure 6A, and the overall structural parameters computed from the SAXS data are given in Table 3. The molecular mass and the volume of the complex exceed those of free CaM by about 14%. This increase is fully compatible with that expected (12%) from the sequences of CaM and the peptide, which proves the formation of a 1:1 complex. On the other hand, the maximum diameter of the complex is practically unchanged, and its radius of gyration is even smaller than that of free CaM. This suggests that CaM keeps the extended conformation in the complex and that the peptide is bound to the central part of CaM, rather than to its periphery. The scattering curve from the atomic model of CaM was computed by the program CRYSOL [29]. It agrees reasonably with the experimental data showing only some minor deviations (Figure 6A, Table 3), which may be attributed to incompleteness of the crystallographic model.

**Table 3:** Structural parameters defined from the experimental SAXS data. *I*_0_, *R*_*g*_, *V*_*p *_and *D*_*max *_are, respectively, the forward scattering, radius of gyration, excluded volume and maximum size calculated from the scattering data. χ_s _and χ_rb _are discrepancies between the experimental data and computed scattering curves from typical *ab initio *and rigid body models (crystallographic model in case of free calmodulin).

Sample	I_0_, relative	R_g_, nm	V_p_, nm^3^	D_max_, nm	χ_s_	χ_rb_
Calmodulin	95.0 ± 5	2.22 ± 0.05	34 ± 2	7.0 ± 0.5	0.8	2.4
Calmodulin+peptide	108.0 ± 3	2.08 ± 0.05	39 ± 2	7.0 ± 0.5	0.8	1.1
Ratio	1.14	0.94	1.15	1.0	-	-

#### Distance distribution functions

The distance distributions *p(r) *of the two samples computed from the experimental data by the program GNOM [30] are given in Figure 6B. These functions are similar up to intraparticle distances of 1.2 nm but display different profiles at larger distances. The *p(r) *of free CaM has a skewed form characteristic for elongated particles, and the maximum in the *p(r) *function at about 4 nm, corresponding to the distance between the two lobes of CaM, whereas the more symmetric *p(r) *of the complex clearly points to a more globular shape. Taken together, the comparison of distance distribution functions also confirms the complex formation, with the interface at the central part of CaM. A decrease in CaM length upon peptide binding, as observed in a number of structures of high-affinity CaM-peptide complexes [31-33] is not observed. *Ab initio *shapes of free and loaded CaM generated by DAMMIN (not shown) also confirm that there is no significant change in the maximum dimension of CaM upon peptide binding.

#### Shape determination

**Figure 7:**
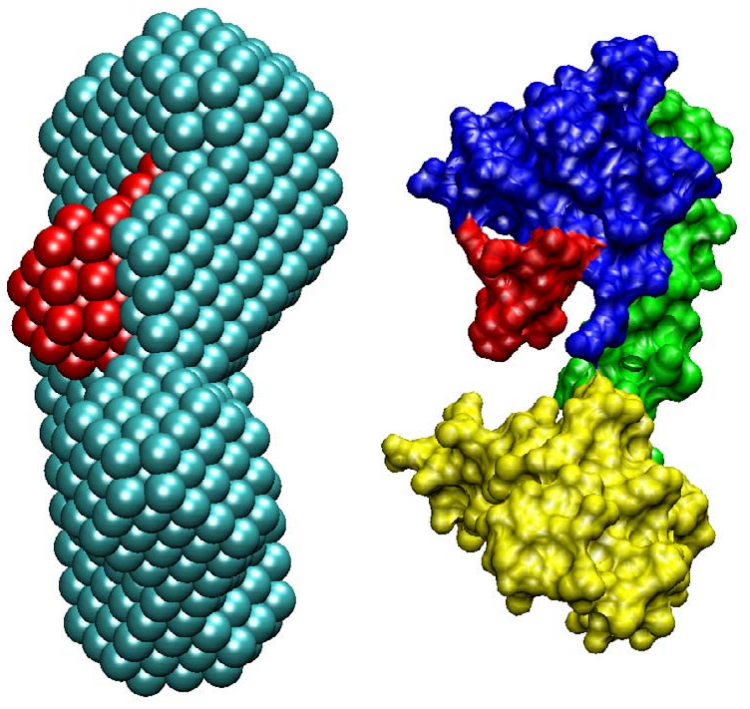
***Ab initio *and rigid body models of the complex**. Left panel: two-component shape of the complex generated by MONSA; Right panel: the rigid body model built by SASREF. The peptide parts are given in red. The central helix and the N- and C-terminal lobes of CaM in the rigid body model are shown in green, yellow, and blue, respectively. The handedness of the *ab initio *MONSA model was chosen that provides the best agreement (lowest NSD) with the rigid body model.

The *ab initio *model of the complex highlighting the location of the peptide was generated by the multiphase bead modeling program MONSA [34]. Several independent runs yielded reproducible results, in which experimental scattering profiles were neatly fitted (Figure 6A, Table 3). The models created by MONSA were very similar to those generated by DAMMIN independently for free and loaded CaM. This finding supports the assumption that no significant change of overall shape occurs upon binding and the use of the two curves for simultaneous fitting by MONSA is valid. A typical model selected by DAMAVER [35] presented in Figure 7 yielded an average normalised spatial discrepancy (NSD) value below 1.0 (see Materials and Methods). It demonstrates that the peptide is positioned between the two lobes of CaM, being closer to one of them. The overall appearance is, thus, in agreement with the conclusions made from primary data processing.

#### Molecular modelling

The crystallographic model of CaM is compatible with the experimental SAXS data from the free protein, and no significant compaction of CaM was observed in the complex. As small conformational changes upon peptide binding could still not be excluded, we split CaM into three fragments (central helix, N- and C-termini of CaM) for the molecular modelling of the complex. The peptide was modelled as a helix for simplicity and due to homology, but the low resolution of the SAXS data does not allow for the determination of the exact conformation of the peptide. The program SASREF [36] was employed to construct a low-resolution 3D model of CaM loaded with the peptide by using distance restraints from NMR data and requirement of chain compatibility for the three CaM segments (see details in Methods). This approach assumes that the CaM residues with the largest chemical shift perturbations in the NMR experiment interact closely with the peptide, which may not be strictly true for all of them. A typical rigid body model of the complex generated in multiple SASREF runs (Figure 7) yields a good fit to the experimental data of the complex (Figure 6A, Table 3). The overall shape of CaM in the rigid body modelling remains similar to that of free CaM. Similarly to the *ab initio *modelling results, the results suggest that the peptide is located between the two lobes, and more specifically, the tighter contact between the peptide and the C-terminal lobe observed by NMR is compatible with the SAXS results. It should be mentioned that at the given resolution, the models with antiparallel orientations of the peptide-helix are not distinguishable from each other, and solutions with both peptide directions were obtained, yielding the same fit to the SAXS data.

### Size exclusion chromatography

**Figure 8:**
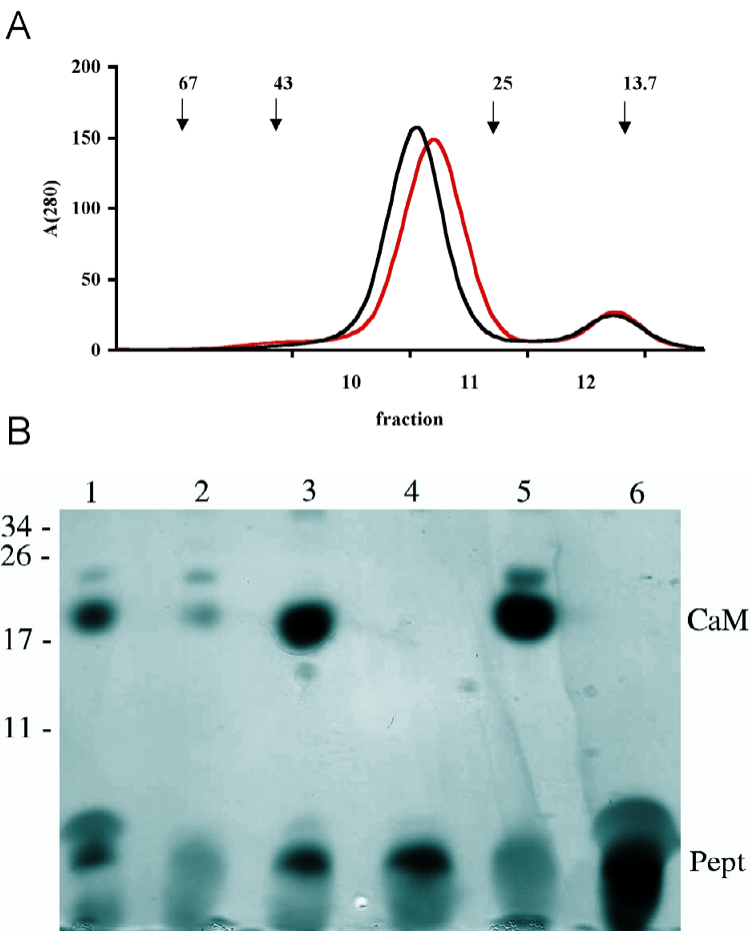
**Gel filtration of CaM in the presence and absence of the MBP peptide**. A. The elution profiles of CaM (black) and the complex of CaM and peptide (red) from a Superdex 75 column. The arrows above indicate the elution volumes and molecular weights (in kDa) of the standard proteins used to calibrate the column. The positions of the collected fractions from the complex sample are indicated below the graph. The absorbances have been multiplied by 1000 for clarity. B. Electrophoretic analysis of the fractions obtained from the complex sample on a 16.5-% Tris-tricine peptide gel. The samples are as follows: 1, the CaM-peptide mixture injected into the column; 2, fraction 10; 3, fraction 11; 4, fraction 12; 5, CaM; 6, peptide. The positions of molecular weight markers (in kDa) are indicated on the left, and the positions of CaM and the peptide are indicated on right. Note how the peak fraction (lane 3) contains both CaM and the peptide. A weak low-molecular-weight shadow is also seen in the CaM sample (lane 5), but the peptide stains intensely dark with silver and can unequivocally be detected based on its colour. The faint band above CaM is most likely another conformation of CaM.

To further validate the determined solution structure of the complex, we carried out gel filtration chromatography and analysed the fractions by SDS-PAGE and silver staining, allowing the visualisation of both CaM and the peptide (Figure 8). Due to its highly extended conformation, free CaM routinely runs at an apparent molecular weight of approximately 30 kDa in gel filtration. A superposition of the chromatograms (Figure 8A) in the presence and absence of the peptide indicates that the hydrodynamic radius of CaM is indeed decreased in the presence of the peptide. An analysis of the collected fractions clearly shows that the large, slightly shifted, peak contains both CaM and the peptide (Figure 8B).

## Discussion

### Native human brain MBP and a peptide from the MBP C-terminus bind CaM

An analysis of native human brain MBP indicated that it interacts directly with CaM in a calcium-dependent manner. A calcium-dependent interaction between human MBP and CaM has previously been reported for two MBP charge isoforms isolated from a patient suffering from multiple sclerosis [20], as have calcium-dependent interactions between bovine and murine MBP and CaM [17-19].

The human brain MBP sample contained isoforms of 17.2 and 18.5 (predominant) kDa after calmodulin affinity chromatography. Thus, the regions encoded by neither exon II (missing in both isoforms) nor exon Vb (missing in the 17.2-kDa isoform) are required for the interaction. Only these two major isoforms are expressed in adult brain [37,38]. The two other major isoforms, 20.2 and 21.5 kDa, are expressed during myelination, as well as during remyelination in MS lesions [39].

A CaM-binding site of MBP has been localised to the C-terminal region, but so far, a detailed mapping has been lacking [20,21]. By the use of various bioinformatics methods and sequence comparisons to known CaM ligands, we were able to design a 19-residue peptide based on the sequence of human MBP that binds to CaM with micromolar affinity. The measured affinity for the peptide towards CaM was similar or slightly weaker than observed previously for full-length MBP and a C-terminal proteolytic fragment thereof [18,21]. Although the observed affinity, in the low micromolar range, is lower than for some other CaM targets, it suggests a physiological relevance for the interaction, taking into account the following points: Firstly, the concentration of MBP in myelin is very high, and the concentration of CaM in cells is also in the order of 1–10 μM [40], even higher in the nervous system [41]. Thus, the measured affinity is in the range that can regulate the interactions between these two highly abundant proteins. Secondly, it is likely that the surrounding regions of MBP, being highly positively charged, can interact with and possibly even wrap around CaM, once the specific recognition of the binding site has occurred; this would be expected to increase the affinity for complex formation. Furthermore, the presence of more than one binding site for CaM in MBP raises the possibility of so-called allovalency [42], effectively increasing the affinity.

It should be noted that in other vertebrate species, insertions of 1–2 residues are seen near the middle of the peptide that was used (see Methods). This implies either that there may be subtle species-specific differences in the interaction between CaM and this MBP segment, or that the entire length of the peptide is not necessary for the interaction. For example, it is possible that only one half of the used MBP peptide is in close contact with the C-terminal lobe of CaM; high-resolution structural data would be required to fully address this issue.

The region, from which the peptide was selected, is also a target for MBP posttranslational modifications. Ser151 can be phosphorylated [43] and Gln147 deamidated [44]. Both of these modifications will add a negative charge to the corresponding position, thus likely decreasing the affinity for CaM. No citrullination sites are present in the peptide, however, due to the lack of arginine residues. On both sides of the used peptide, the MBP sequence does harbour characterised citrullination sites [45], which could, in principle, also regulate binding to CaM.

### Energetics of the CaM-MBP peptide interaction

The heat capacities of several CaM-associated reactions have been reported and discussed earlier [46]. Temperatures below +30°C have been systematically used for ITC binding studies with CaM and CaM-binding peptides. This is important in order to prevent temperature-induced conformational changes in CaM [27,47]; this effect is also seen in our data, making measurements at high temperatures unreliable. CaM-binding peptides have been roughly divided into two groups depending on the heat capacity upon their complex formation with CaM [46]. CaM-dependent protein kinase I, constitutive cerebellar nitric-oxide synthase, and melittin peptides have *ΔC*_*p *_values around -0.8 kcal mol^-1 ^K^-1^, which reflects their preference for the formation of a globular CaM-peptide complex. The second group, having a *ΔC*_*p *_of -0.4 kcal mol^-1 ^K^-1^, is composed of phosphodiesterase, the C-terminal fragment of melittin, and chicken gizzard caldesmon peptides, which interact mainly with one lobe of CaM [46]. In our current study, the estimated *ΔC*_*p *_of the formed complex is -0.13 kcal mol^-1 ^K^-1^, which is different from the above cases and can indicate different type of binding compared to the two previously discussed groups of peptides. In fact, it directly suggests a smaller buried surface area than in the other CaM-peptide complexes – an idea which is indeed confirmed by our NMR and SAXS studies.

### The structure of the CaM-MBP peptide complex in solution

SPR indicated that the complex formed between immobilised CaM and the MBP peptide underwent a conformational change upon binding. A conformational change was also detected by SPR when the complex between CaM and the ERR3 receptor was studied [48]. A negative change in RU, as observed here for the CaM-MBP complex, during the injection may reflect a decrease in the hydrodynamic radius of the forming complex [28]. This is also in line with our results from gel filtration chromatography, in which the apparent size of CaM is decreased in the presence of the peptide, indicating a smaller hydrodynamic radius for the complex than for unliganded CaM. On the other hand, as expected, CD spectroscopy clearly indicated that no large-scale changes in secondary structure content occur upon binding.

The solution structure of the CaM-MBP peptide complex obtained with SAXS is compatible with all of the other experiments we carried out on the complex. In essence, the SAXS results indicate that the maximum diameter of CaM does not change when bound to the MBP peptide, and the radius of gyration for the complex is slightly smaller than that of free CaM. This result is fully compatible with the result from gel filtration chromatography. The peptide can be seen bound to a groove in the central region of CaM (slightly closer to the C-terminal lobe), which remains in the extended conformation. In other words, CaM does not collapse around the MBP peptide, as seen in several other instances of CaM-peptide recognition [31-33].

It is possible that the CaM-MBP peptide complex has several conformations in solution; however, the fact that both the *ab initio *and rigid body models from SAXS fit the measured data well (Figure 6) indicates that it is likely that only one major conformation is present, at the resolution that has been used in the current study. This is also supported by gel filtration chromatography (Figure 8), where significantly different conformations of the complex could yield different peaks or peaks with non-symmetric shapes, since the starting point is CaM, an elongated dumbbell-shaped molecule.

**Figure 9:**
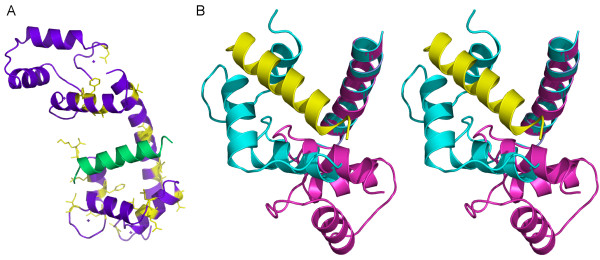
**Mapping of the binding site of the human MBP peptide in CaM to the C-terminal lobe. It should be noted that the structures shown are extrapolations from the low-resolution SAXS model obtained by rigid-body fitting, as described in Methods**. A. CaM (magenta) and the peptide (green) are shown as ribbons, and the interacting residues, based on the NMR experiment, are shown in stick mode with yellow colour. Note how the binding site is localised mainly to the hydrophobic pocket of the C-terminal (lower) lobe. B. Stereo view of the conformation of the CaM C-terminal lobe in free CaM (pink) and in the SAXS model of the complex (light blue). The peptide is shown in yellow, and superposition has been done using the residues of the central helix (pointing towards top right). The N-terminal lobe is omitted from the figure for clarity. An animation of the predicted movement is shown in Additional file 2.

Our NMR data indicate that although the main binding site for the MBP peptide resides in the C-terminal lobe of CaM, also the central helix and the N-terminal lobe interact with the peptide. Comparing the structures of our model and free CaM, the NMR data suggest that a rotation of the C-terminal lobe takes place upon binding, such that its hydrophobic pocket interacts closely with the peptide (Figure 9, Additional file 2). Furthermore, it should be noted that molecular modelling was used in an earlier study to predict the modes and energetics of binding of different short MBP peptides to CaM, including one overlapping with the peptide used in this study [22]. As a starting point in these simulations, the fully collapsed CaM structure from a kinase peptide complex was used. Our results indicate that this may have been an oversimplification, and that the conformation of CaM may be significantly different in the CaM-MBP complex.

For CaM-dependent protein kinases, it has been suggested that studies on CaM-binding peptides accurately reflect the interactions between CaM and the kinase [49]. It is possible that for MBP, regions outside the peptide studied here also participate in complex formation, possibly altering the affinity and/or conformation of the complex, as has been observed for the deiminated form of MBP [20]. Interestingly, it has also been suggested that CaM might be able to bind two molecules of MBP simultaneously in a cooperative fashion, where both lobes of CaM would mainly interact with one MBP molecule [19]. Thus, the structure of the complex determined here may also represent an intermediate in the binding of full-length MBP by CaM.

## Conclusion

Structural knowledge about myelin proteins in general is underrepresented in current databases [50,51], despite the presence of a well-characterised but relatively small set of specific proteins in the myelin sheath. We have carried out a detailed analysis of the interaction between CaM and a 19-mer peptide from the C-terminal region of human MBP, a major protein in the myelin sheath. MBP has been characterised as being intrinsically unstructured by nature [3]; however, it presents a multitude of post-translational modifications and binding partners, which, in turn, affect its functions in *e.g*. signaling and regulating the cytoskeleton [2]. We have shown that the complex formed between CaM and the MBP peptide does not collapse, although a conformational change takes place on a smaller scale, the main binding site for the MBP peptide residing in the hydrophobic pocket of the C-terminal lobe of CaM. With relatively weak binding, such as that observed between MBP and CaM, a massive conformational change may indeed not be required, and the binding affinity may be low in order to maintain the possibility of regulation, because the concentrations of both interaction partners in the cell are high. Thus, MBP is another CaM target that cannot be thought of as being analogous to CaM kinases with respect to the mode of CaM binding [52]. Our results provide an important structural insight into the factors that may regulate myelin formation and maintenance and further highlight the plasticity of CaM in target recognition.

## Methods

### Sequence analysis

A number of sequence analysis tools were applied in order to be able to select a suitable CaM-binding peptide from the C-terminal region of human MBP that has been suggested to carry a CaM-binding site [20,21]. Disorder predictions were carried out using DISembl [53,54], GlobPlot [55,56], and DRIP-PRED [57]. Secondary structure predictions were done using the consensus method at the NPSA [58,59]. Putative functionally flexible regions were analysed with Wiggle [60]. The CaM target database [25,61] was searched for putative CaM-binding segments within MBP, and in the end, the chosen region was manually aligned with CaM-binding regions from CaM-dependent protein kinases. It should be noted that this method is unlikely to detect previously uncharacterised CaM-binding sites.

### Materials

The MBP peptide chosen for the present study, HKGFKGVDAQGTLSKIFKL, corresponding to residues 138–156 of the human MBP 18.5 kDa isoform, of 95% purity, was obtained from Genemed Synthesis. It should be noted that some other vertebrates have 1- or 2-residue insertions in this region, at position 145. The peptide was dissolved in 50 mM HEPES pH 7.5, 20 mM CaCl_2 _and stored at -20°C. The preparation of the CaM expression construct pETCM, containing vertebrate CaM cDNA, has been described earlier [62]. Native MBP protein isolated using the chloroform extraction method [63] from post-mortem human brain was obtained from FermLab (Turku, Finland).

### Protein purification

CaM was purified using calcium-dependent phenyl sepharose affinity chromatography [62,64]. pETCM was transformed into *E. coli *Rosetta (DE3), and one colony was transferred into 10 ml of LB containing 100 μg/ml of ampicillin and grown for 7 h at +37°C. For expression, 1 l of LB medium containing ampicillin (100 μg/ml) was inoculated with the 10 ml culture. Induction was performed after 5 h with 0.4 mM IPTG. The culture was further grown for 12 h, and the cells were harvested by centrifugation. For stable isotope labeling, modified M9 medium [65] was used instead of the LB medium. The cells were suspended in 50 ml of cold lysis buffer (10 mM HEPES pH 7.5, 1 mM EDTA, 1 mM NaN_3_, 10 mM DTT, 10 μg/ml DNAse I), and lysozyme and RNAse A were added to 100 μg/ml and 2 μg/ml, respectively. Cell suspensions were stored at -80°C until needed. Cells were disrupted by sonication. After centrifugation (27000 g, 20 min), the supernatant was heated in a +80°C water bath for 10 min and centrifuged (27000 g, 20 min). 5 mM CaCl_2 _were added to the supernatant, and the sample was centrifuged once more.

The soluble proteins were applied to a phenyl sepharose column (Pharmacia Biotech Inc), equilibrated with 10 mM HEPES pH 7.5, 4 mM CaCl_2_. The column was consecutively washed with excess equilibration buffer, equilibration buffer supplemented with 500 mM NaCl, and equilibration buffer again. Elution of CaM was then carried out with 10 mM HEPES pH 7.5, 5 mM EGTA. The fractions were analyzed by SDS-PAGE, and the fractions containing CaM were pooled and concentrated by centrifugal ultrafiltration.

### Affinity chromatography of human brain MBP on CaM-sepharose

Calcium-dependent affinity chromatography of human MBP on CaM-sepharose (GE Healthcare) was carried out as follows: 200 μg of lyophilized human brain MBP were dissolved in 50 mM HEPES pH 7.5, 150 mM NaCl, 2 mM CaCl_2_. The same buffer that was used to equilibrate 100 μl of the CaM-sepharose matrix. The sample was mixed with the matrix and washed with 5 ml of the above buffer. Elution was done with 1.4 ml of 50 mM HEPES pH 7.5, 150 mM NaCl, 2 mM EGTA. The fractions were applied on a 18% SDS-PAGE gel. To detect which isoforms were bound to CaM-sepharose, fractions 2 (wash) and 7 (EGTA elution) were purified with Vivapure^® ^C18 Micro spin columns prior to analysis by mass spectrometry. MALDI-ToF mass spectrometry was performed with a Voyager-DE STR Biospectrometry Workstation (Applied Biosystems). 1 μl of the protein sample was spotted on sinapinic acid matrix.

### Circular dichroism spectroscopy

Far-UV CD spectra in the 196–250 nm region were recorded at +20°C with a JASCO J-715 CD-spectropolarimeter (Jasco Corp., Tokyo, Japan), using a 1-mm path length quartz cell. The spectra were corrected by subtracting the spectrum of a buffer sample (10 mM HEPES pH 7.5, 150 mM NaCl, 4 mM CaCl_2_). Noise reduction was applied to smooth the data with the Jasco software. The spectra are presented as mean residue ellipticity (θ), calculated by using the molar concentration of peptide or protein and multiplying by the appropriate number of peptide bonds. CaM concentration in the sample was 0.050 mg/ml and the peptide concentration was 0.030 mg/ml. Secondary structure contents of the samples were estimated with the program k2d [26].

Thermal denaturation of CaM in the presence and absence of the MBP peptide was followed by monitoring the ellipticity at a fixed wavelength of 222 nm while the sample was heated at a rate of 30°C/h from +20 to +98°C. Free CaM concentration in the sample was 0.15 mg/ml and in the CaM/peptide mixture, the concentrations of CaM and the peptide were 0.20 and 0.30 mg/ml, respectively. A cooling experiment to monitor putative refolding of the protein was also similarly done for the same samples from +98 to +20°C at a rate of 30°C/h

### Isothermal titration calorimetry

The binding of the MBP peptide to CaM was studied by ITC, using a VP-ITC microcalorimeter (MicroCal, Norhampton, MA). The samples for the experiment were extensively dialysed against 50 mM HEPES pH 7.5, 20 mM CaCl_2 _and degassed in an ultrasonic water bath. The MBP peptide solution (1.0 mM) was injected into the sample cell, containing 0.10 mM CaM in the above buffer. The first injection of 2 μl was followed by 19 15-μl injections of the peptide. The experiment was carried out at +25, +30, +35, and +40°C. The dilution heat was measured by titrating the buffer with the 1 mM peptide solution. The heat of dilution was practically zero. The raw data were fitted by non-linear least squares minimisation with models for both a single site and four binding sites (see Results), using the Origin version 5.0 software (MicroCal).

### Surface plasmon resonance

SPR assays were performed with a Biacore 3000 instrument (Biacore AB, Uppsala, Sweden). CaM was immobilized covalently on a hydrophilic carboxymethylated dextran matrix sensor chip (Biacore AB), with standard amine coupling chemistry [66] at pH 3.8, at a level of 4000 RU. Binding of the peptide was tested in 10 mM HEPES pH 7.5, 150 mM NaCl, 4 mM CaCl_2 _containing 0.005% P-20 surfactant (Biacore AB), at a flow rate of 30 μl/min. Various concentrations of the peptide (2.5–200 μM) were injected in duplicate over both of the flow cells for 3 min, and a 6-min dissociation phase followed each injection. The surfaces were regenerated with a 20-s injection of 10 mM EDTA and another 10-s injection of 20 mM NaOH at a flow rate of 30 μl/min prior to the next injection cycle. The BIAevaluation software version 3.1 (Biacore AB) was used to analyse the data. Bulk effects were subtracted using a reference surface without CaM. Furthermore, a sensorgram of the injection of buffer alone was subtracted from the data. The data were analysed using the various interaction models available in the software, including the mass transfer model. The only model giving a reasonable fit to the data was the conformational change model (see Results).

A control experiment for linked reactions ("heterogeneity test") for the binding reaction was used to analyse the stability of the forming complex as a function of injection time. A 20-μM solution of the peptide in the running buffer was injected over the flow cells of the CaM-coated sensor chip at a flow rate of 30 μl/min, using different injection times (1, 3, and 20 min). Each injection was followed by a 15-min dissociation time. The surface was regenerated and the data were treated as above.

### Small-angle X-ray scattering

#### SAXS data collection and processing

Synchrotron X-ray scattering data from solutions of calmodulin in the presence and absence of the MBP peptide were collected at the X33 beamline (DESY, Hamburg) using a MAR345 image plate detector. The scattering patterns of both samples were measured at several solute concentrations *c*, ranging from 5.4 to 140.0 mg/ml, and in the complex samples, a 5-fold molar excess of the peptide was always present. For the complex, it was calculated, based on the determined K_d _from calorimetry, that more than 99.8% of total CaM was expected to be in the complex state even at the lowest used concentration. At a sample-detector distance of 2.7 m, the range of momentum transfer 0.1 <*s *< 5 nm^-1 ^was covered (*s *= *4π sin(θ)/λ *where *2θ *is the scattering angle and *λ *= 0.15 nm is the X-ray wavelength). The data were processed using standard procedures by the program package PRIMUS [67]. The forward scattering *I(0) *and the radii of gyration *R*_*g *_were evaluated using the Guinier approximation [68], assuming that at very small angles (*s *< 1.3/*R*_*g*_), the intensity is represented as *I*(*s*) = *I(0) exp(-(sR_g_)^2^/3)*. The maximum dimensions *D*_*max *_were computed using the indirect transform package GNOM [30], which also provides the distance distribution functions *p(r)*.

The increase in the molecular mass (MM) of CaM upon peptide binding was verified by the analysis of the forward scattering value *I(0) *using the proportion MM ~ *I(0)/c*. In addition, the excluded (Porod) volumes *V*_*p *_of the solutes were also analyzed, calculated as [69]:

$$V=2\pi^{2}I(0)/\int_{0}^{\infty }s^{2}I(s)ds$$

In this calculation, an appropriate constant was subtracted from each data point to force the *s*^-4 ^decay of the intensity at higher angles following Porod's law [69] for homogeneous particles. For globular proteins, Porod (*i.e*. hydrated) volumes in nm^3 ^are approximately twice the MMs in kDa.

#### Ab initio shape determination

Low resolution *ab initio *shapes of free and loaded CaM were first reconstructed independently using the program DAMMIN [34]. In a more versatile approach, the *ab initio *model of the complex was constructed by simultaneous fitting of the scattering from free and loaded CaM by a two-component model using the program MONSA [34,70]. The program represents the particle as a collection of M >> 1 densely packed beads inside a sphere with the diameter *D*_*max*_. To describe the overall and internal structure of the complex particle, each bead can be assigned either to the solvent (index = 0) or to one of the components (in our case, index = 1 corresponds to CaM and index = 2 to the MBP peptide). The complex between CaM and the peptide is therefore represented at low resolution by two "phases", and the structure is described by a string of length M, containing the phase index for each bead (0, 1, or 2). Simulated annealing (SA) [71-73] is employed to search starting from a random string for a model composed by interconnected compact phases, which simultaneously fits the shape scattering curves from free CaM and the complex (*I*_*k*_(*s*), k = 1, 2) to minimize overall discrepancy:

$$\chi^{2}=\sum_{k}\frac{1}{N_{k}-1}\sum_{j}{\left[\frac{I_{k}(s_{j})-c_{k}I_{k}^{calc}(s_{j})}{\sigma_{k}(s_{j})}\right]}^{2}$$

where the index *k *runs over the scattering curves, *N*_*k *_are the numbers of experimental points, *c*_*k *_are scaling factors, and *I*_*calc*_(*s*) and *σ*(*s*_*j*_) are the intensities calculated from the subsets of the beads, belonging to the appropriate phases and the experimental errors at the momentum transfer *s*_*j*_, respectively.

#### Molecular modelling

SASREF [36] was employed for molecular modelling of the complex, using the available atomic model of vertebrate calcium-loaded CaM (PDB entry 1UP5) [74]. SASREF employs SA to minimize the target function *F *in a form:

$$F=\chi^{2}+\sum_{i}\alpha_{i}P_{i},$$

where the first term ensures minimization of the overall discrepancy and the set of penalties *P*_*i *_with their weights α_i _formulates various requirements and restrictions to the model (*e.g*. interconnectivity, contacts absence of overlaps) [36]. The penalty weights for disconnectivity and distance restraints had the default values of 10. The weight of the overlap penalty was increased (to 100), as the resulting models tend to have some clashes while using the default weight.

The peptide was modelled by modifying the sequence of the homologous peptide from human DRP-1 kinase, as present in the corresponding CaM-peptide complex (PDB entry 1WRZ). To account for possible conformational changes upon peptide binding, CaM was represented by three rigid bodies, namely by the N-terminal lobe, the central helix, and the C-terminal lobe (residues 4–64, 65–93, and 94–148, respectively). SASREF positions the four rigid bodies (three CaM portions and the peptide) with respect to each other using SA to form an interconnected assembly without steric clashes, while minimising the discrepancy between the experimental data and the scattering profile from the model of the complex. The latter is calculated based on the pre-computed scattering amplitudes of the subunits in the reference positions and orientations. Scattering from the atomic models was calculated using the program CRYSOL [29]. SASREF allows one to account for contacts between specific residues in the subunits using distance restraints. In the present work, the polypeptide chain compatibility of the entire CaM was ensured by restraining the distances between appropriate terminal residues of three CaM portions to 0.4 nm. Moreover, the information on chemical shift perturbations from the NMR experiment (see NMR section) was also included into SAXS-based molecular modelling by adding the proximity requirement between the peptide and the CaM residues that were observed to be involved in binding the MBP peptide. In brief, a distance restraint of 10 Å was used between the peptide and residues 78, 80, 85, or 88 of CaM. A restraint of 7 Å was imposed on the distance between the peptide and CaM residues 130, 137, or 141 and between the peptide and CaM residues 143 or 147. The restraint terms were not adjusted during minimisation. A discussion on the shape of the restraint functions has been previously published [36].

For the *ab initio *and rigid body analyses, multiple runs were performed to verify the stability of the solution, and typical 3D reconstructions are presented in the results section. The models generated in 10 independent runs yielded an averaged NSD value of about 1.0 according to the automated superposition program SUPCOMB [75]. Such an NSD value means that the models are indistinguishable at the resolution provided by SAXS. The analysis of common structural features of the models was done using the programs DAMAVER [35] and SUPCOMB. The latter program aligns two arbitrary low or high resolution models represented by ensembles of points by minimizing a dissimilarity measure, NSD. For every point (bead or atom) in the first model, the minimum value among the distances between this point and all points in the second model is found, and the same is done for the points in the second model. These distances are added and normalized against the average distances between the neighboring points for the two models. Generally, NSD values close to unity indicate that the two models are similar. The program DAMAVER specifies the most typical model (i.e. that having the lowest average NSD with all the other models of the set of superimposed structures).

The rigid body model and the original data are included in this paper as Additional files 3 and 4, respectively.

### Size exclusion chromatography

Gel filtration chromatography with the ÄKTA*purifier *system (Pharmacia Biotech) was used to investigate the difference in the behaviour of CaM in the presence and absence of the MBP peptide. A Superdex 75 HR 10/30 column (Amersham Pharmacia Biotech) was equilibrated with 50 mM HEPES buffer pH 7.5, 150 mM NaCl, 4 mM CaCl_2_. The experiments were run at a flow rate of 0.5 ml/min, the injection volumes were 250 μl, the concentration of CaM was 0.8 mM, and the concentration of the peptide was 4 mM. SDS-PAGE on a 16.5% Tris-tricine gel (Bio-Rad) combined with silver staining [76] was used to visualise the peptide and CaM from the fractions.

### Nuclear magnetic resonance spectroscopy

All NMR experiments were carried out on a Bruker DMX-500 spectrometer using a 5-mm broadband, z-axis gradient-shielded probe at 298K. Two-dimensional ^1^H-^15^N HSQC spectra were obtained by pulse field gradient selection [77]. The sweep width was 12 ppm in the ^1^H dimension and 30 ppm in the ^15^N dimension, with the ^1^H carrier set at 500.1324 MHz and the ^15^N carrier at 50.6814 MHz. The size of the HSQC spectra was a 1024 × 1024 real data matrix with eight scans for each experiment. Proton chemical shifts were referenced to 2,2-dimethyl-2-silapentane-5-sulfonate as 0 ppm. Nitrogen-15 chemical shifts were referenced to liquid NH_3_.

The sequential assignments for the backbone nuclei of the ^15^N/^13^C-labeled Ca^2+^/CaM were mainly achieved by a set of experiments, CBCA(CO)NNH, CBCANNH, HNCO, and HN(CA)CO. Sidechain assignments were obtained using HBHA(CO)NNH, H(CCCO)NNH, CC(CO)NNH, and HCCH-TOCSY experiments. The assignments for the protein backbone nuclei of the ^15^N-labeled Ca^2+^/CaM – MBP peptide complex were achieved by the two-dimensional ^1^H-^15^N HSQC spectral changes induced by titrations of myelin peptide to the Ca^2+^/CaM. NMR spectra were processed on a Silicon Graphics O2 workstation using Bruker XWIN-NMR and Accelrys Felix 2000 software packages.

## Abbreviations

CaM, calmodulin; MBP, myelin basic protein; CD, circular dichroism; ITC, isothermal titration calorimetry; SPR, surface plasmon resonance; SAXS, small-angle X-ray scattering; SA, simulated annealing; CNS, central nervous system; PNS, peripheral nervous system; RU, resonance unit; NMR, nuclear magnetic resonance; MM, molecular mass; NSD, normalised spatial discrepancy

## Authors' contributions

PK and VM conceived of the study. All authors participated in experiments, data analysis, and writing the paper, and have read and approved of the final draft.

## Supplementary Material

Additional file 1 from CaM-sepharose chromatography. Mass spectroscopic analysis of fractions 2 (2nd wash, top) and 7 (2nd EGTA eluate, bottom) from affinity chromatography of human brain MBP on CaM sepharose. Note the presence of the 17.2- and 18.5-kDa forms of MBP in both fractions. Click here for file

Additional file 2 Conformational change of the C-terminal lobe of CaM upon peptide binding. A stereo Quicktime movie showing the predicted movement occurring in the C-terminal lobe of CaM upon binding the MBP peptide. The conformation of the complex is as obtained by the combined use of our NMR and SAXS data. Colouring as in figure 9B. Click here for file

Additional file 3 The model refined by SASREF rigid body fitting against the SAXS data, including restraints based on the NMR results. Click here for file

Additional file 4 The measured SAXS data; buffer blank scattering has been subtracted. Click here for file
