# Supplementary figures and images for: Interaction between the C-terminal region of human myelin basic protein and calmodulin: analysis of complex formation and solution structure
Source: BMC Struct Biol. 2008 Feb 19;8:10. doi: 10.1186/1472-6807-8-10 (PMC2288786; doi:10.1186/1472-6807-8-10)

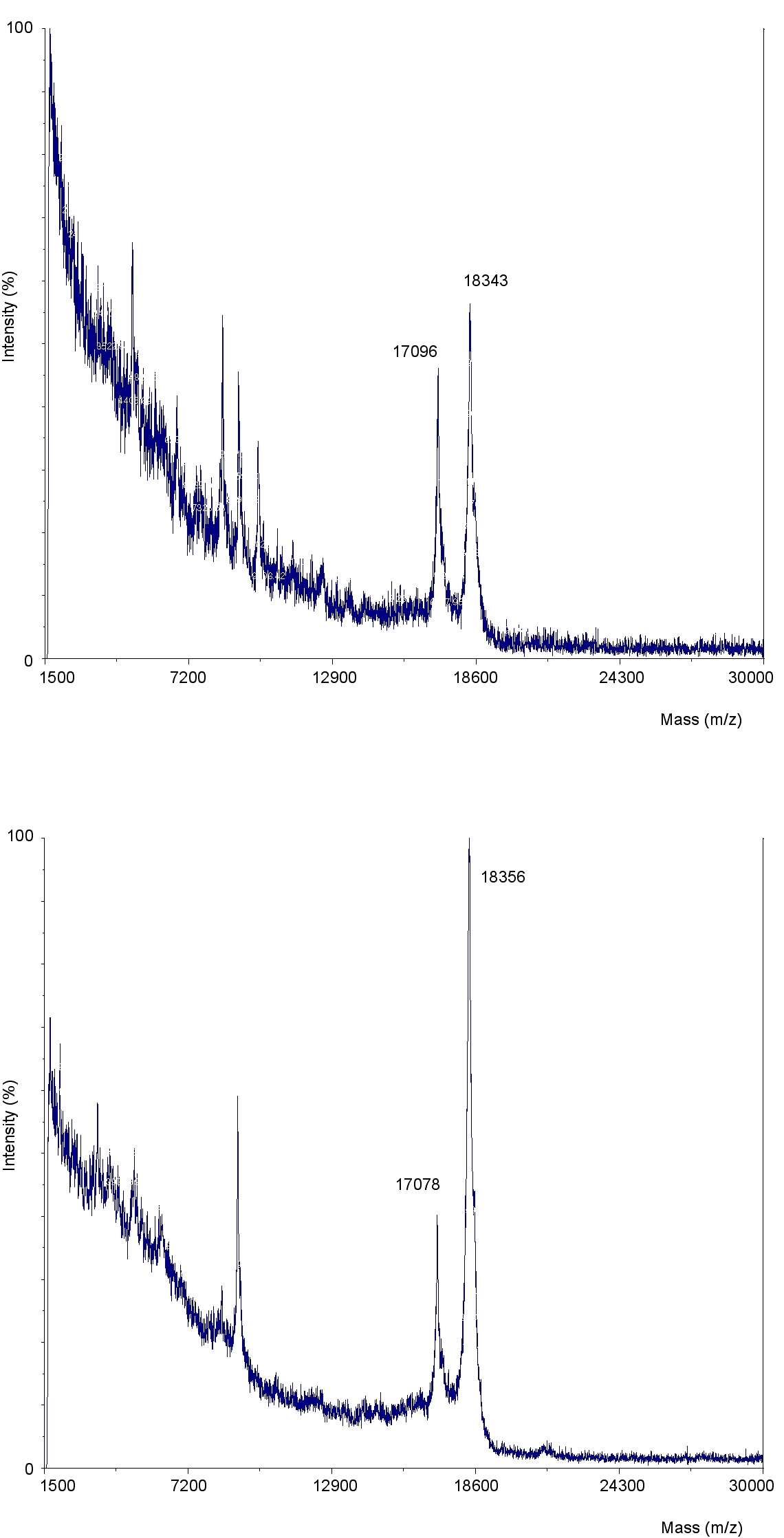

Supplement: Additional file 1 — from CaM-sepharose chromatography. Mass spectroscopic analysis of fractions 2 (2nd wash, top) and 7 (2nd EGTA eluate, bottom) from affinity chromatography of human brain MBP on CaM sepharose. Note the presence of the 17.2- and 18.5-kDa forms of MBP in both fractions. [file 1472-6807-8-10-S1.jpeg]
